# Sleep quality and fatigue level, clinical and demographic factors in women with rheumatoid arthritis

**DOI:** 10.3389/fgwh.2025.1534447

**Published:** 2025-06-05

**Authors:** Katarzyna Anna Kozłowska, Grażyna Bączyk

**Affiliations:** Department of Nursing Practices, Poznan University of Medical Sciences, Poznan, Poland

**Keywords:** fatigue, rheumatoid arthritis, clinical factors, demographic factors, quality of sleep

## Abstract

**Background:**

Sleep, as one of the factors influencing people's lives, strongly impacts the standard of living of RA (rheumatoid arthritis) patients. The study aimed to determine the level of sleep quality in RA women and whether fatigue and selected demographic and clinical factors influence it.

**Methods:**

The study included 110 female patients diagnosed with RA. Sleep was assessed using The Pittsburgh Sleep Quality Index (PSQI), Functional Assessment of Chronic Illness Therapy-Fatigue (FACIT-F) was used to evaluate fatigue, and a proprietary questionnaire was used to assess demographic and clinical factors.

**Results:**

Most women had low sleep quality (>5 points)—71.1% of people. Poor sleep quality in older people (*p* = 0.0123) and married patients (*p* = 0.0367). Poor sleep quality was also influenced by pain (*p* = 0.0006), morning stiffness (*p* = 0.0002), DAS28 (*p* = 0.0367), and feeling of fatigue (*p* < 0.0001). In multiple and logistic regression analysis, pain was the main factor contributing to poorer sleep quality.

**Conclusion:**

Assessment of factors influencing sleep quality that influence RA patients’ well-being is very important. Determining which factors affect the quality of life will allow them to be controlled and mitigated. Our research has shown that pain is primarily responsible for reducing sleep quality.

## 1 Introduction

Rheumatoid arthritis (RA) is an autoimmune disease involving inflammation of symmetrical joints, also causing complications in other organs. It is characterized primarily by pain in the affected joints, deformity, and morning stiffness. It is an incurable disease with periods of remission and exacerbation. Patients affected by RA show signs of disability, which significantly affects their quality of life and mental state and hurts the social, private, and professional spheres of life (1–3).

Many aspects negatively or positively impact the well-being of RA patients. One of them is sleep. Sleep, oxygen, water, and food are essential for life. Its disorders, such as ineffectiveness, being too short or too long, difficulty falling asleep, and frequent waking up, negatively affect a person's health, lifestyle, fulfillment of social roles, or professional work in healthy people. Poor sleep quality in RA patients may be caused by pain, changes in the respiratory system (causing sleep apnea, insomnia), depression, or fatigue. Paradoxically, sleep abnormalities worsen the physical and mental condition of the sick person. They also associated the ineffectiveness of treatment with deepening the symptoms and increasing the disease's activity (4–6).

Many studies in recent years have shown that fatigue is a factor that strongly impacts the sense of well-being of RA patients. Fatigue is a symptom that causes severe inability in patients to undertake any activity and is characterized by a lack of vigor and a feeling of exhaustion. One of the determinants that intensifies it is sleep. Feeling tired causes problems with falling asleep, and poor sleep quality deepens the feeling of tiredness during the day (7–10).

The study aimed to determine how fatigue and clinical and demographic factors affect sleep quality in women with RA. This is extremely important because the literature review shows great diversity in this area, depending on the studied population. To a small extent, several factors’ influence on the sleep quality level in RA patients has been analyzed simultaneously. Therefore, this work focused on the assessment of the impact of several factors jointly on the level of sleep quality, which was carried out here (logistic and multiple regression), and an indication of whether several or one factor had a greater impact on the assessment of its level. It is also essential to indicate that confounding factors influence the evaluation of sleep quality, e.g., the time of completing the questionnaires after the treatment was implemented, lack of treatment for any symptoms (e.g., anemia), or omission of treatment by the patients.

## 2 Materials and methods

### 2.1 Study and participants

The study group consisted of 110 women admitted to the rheumatology department in the Poznan Hospital. The data study was collected in 2016–2018. That study is part of a bigger project (9, 11).

Patients diagnosed with RA according to the European League Against Rheumatism (EULAR) in 2010 criteria (12) who had the necessary laboratory test results and understood the meaning of the questions asked were eligible for the study. The exclusion criterion was the occurrence of diseases associated with increased fatigue intensity, such as malignancy and mental illness. An additional exclusion criterion was the lack of consent or resignation during the investigation and failure to meet any of the above points. Study participants were asked to complete the questionnaires independently or with the researcher's participation (whose role was to read the questions and provide answers when the patients could not do it themselves).

### 2.2 Methods/questionnaires used

Two standardized questionnaires and an authoring tool were used to achieve the study's objectives. All respondents completed the same questionnaires.

The Pittsburgh Sleep Quality Index (PSQI) questionnaire assessed sleep quality. The PSQI questionnaire consists of 10 questions about patients’ typical sleep habits over the last four weeks. It evaluates a broad range of sleep quality parameters such as sleep hygiene, difficulties falling asleep, problems maintaining sleep continuity, and daytime functioning. It also includes questions about the causes of sleep disturbances, including questions directed to the person sleeping in the same room as the patient. Responses are rated on a Likert scale. The minimum score that can be obtained is 0 points, and the maximum is 21 points, where a higher score indicates poorer sleep quality. A score above five points indicates reduced sleep quality (13).

Functional Assessment of Chronic Illness Therapy-Fatigue (FACIT-F) version 4 (consists of 13 questions) assesses the severity of fatigue symptoms. This scale lets measures the level of fatigue in physical, mental, and social areas during last week. The respondent assesses the impact of fatigue on the daily functioning and the related activities of daily living observed. A minimum of 0 points, the maximum is 52 points (the higher the score, the less severe the symptoms of fatigue) (14).

A proprietary questionnaire was used to assess demographic factors and part of clinical factors, containing questions regarding age, disease duration, current treatment [classic nonsteroidal anti-inflammatory drugs (NSAIDs), disease-modifying drugs, glucocorticosteroids], biological treatment (yes/no), the hemoglobin concentration (Hgb), CRP (C-reactive protein), rheumatoid factor (RF) titer, and asked about the duration of the morning stiffness symptom (in minutes). DAS28 was used to assess other clinical variables, the Visual Analogue Scale (VAS) scale (0–10 cm) was used to determine the severity of joint pain, with a scale used to determine joint tenderness and swelling the Ritchie Articular Index.

### 2.3 Statistical analysis

Qualitative variables were described using number (*n*) and frequency (%), and measurable variables were defined using the arithmetic mean, standard deviation (std. dev.), median, and minimum and maximum values (min. and max.).

The Pearson chi-square test was used to examine the relationships between qualitative variables, and in the case of too small expected numbers in a 2 × 2 contingency table, the chi- square test with Yates’ correction was used, and in a contingency table larger than 2 × 2, the NW chi-square test was used (the most considerable credibility). Fisher's exact test was used for a 2 × 2 contingency table with a count of less than 40.

Due to the nature of the variables (discrete, measurable variables and lack of normality of distribution), non-parametric tests were used for statistical analyses: the Mann–Whitney *U* test (to check the significance of differences in two groups) and the Spearman's rank correlation coefficient significance test (to examine the correlation between measurable variables).

The t-test was used to check the significance of differences in the two groups if the normality of distribution and the homogeneity of variance were met.

For multivariate analysis, multiple regression analysis was used for PSQI as a measurable variable, and logistic regression was used for PSQI as a variable with two states: low (>5 points) and good (≤5 points) sleep quality.

The size calculation is *n* = 96, with a confidence level of 0.95 (*α* = 0.05) and an assumed maximum error of 10%.

A value of *p* < 0.05 was considered statistically significant. Statistical calculations were performed using the STATISTICA 10 PL statistical package.

## 3 Results

### 3.1 Characteristics of the study group

The study group consisted of 110 female patients with RA. The respondents were between 19 and 83 years of age; the average age for women was 53.7 ± 14.4. Only 19 females (17.3%) were treated with biological drugs, the remaining patients (82.7%) were treated with disease modifying antirheumatic drugs and corticosteroids (14). General characteristics of participants in terms of their clinical data and demographic variables showed the Table 1.

**Table 1:** General characteristics of participants in terms of their demographic variables and clinical data.

Demographic variables	Female *n* = 110 (85.9%)	
	*n*	%
Place of residence		
City	77	70.0
Country	33	30.0
Marital status		
Single	21	19.1
Married	72	65.5
Divorced	4	3.6
Widowed	13	11.8
Education		
Elementary school education	4	3.6
High school education	73	66.4
Higher education	33	30.0
Occupational status		
Professionally active	53	48.2
Unemployed	5	4.5
Retired	35	31.8
Pensioner	17	15.5
Residence status		
With someone	96	87.3
Alone	14	12.7
Biological treatment		
Yes	19	17.3
No	91	82.7
Age [years]		
Avg. ± SD	53.7 ± 14.4	
Median (min.–max.)	56.0 (19–83)	
Clinical variables	Total *n* = 110	
Disease duration [years]		
Avg. ± SD	11.1 ± 8.7	
Median (min.–max.)	9.5 (0.2–40)	
Visual analogue scale for pain (VAS) [cm]		
Avg. ± SD	5.6 ± 2.4	
Median (min.–max.)	6.0 (0–10)	
Morning stiffness [min]		
Avg. ± SD	53.3 ± 69.1	
Median (min.–max.)	30.0 (0–420)	
Hemoglobin (Hgb) [g/dl]		
Avg. ± SD	9.3 ± 2.8	
Median (min.–max.)	8.4 (3.4–23.2)	
C-reactive protein (CRP) [mg/L]		
Avg. ± SD	11.5 ± 15.6	
Median (min.–max.)	5.9 (0.1–84.4)	
Rheumatoid factor (RF) [IU/ml]		
Avg. ± SD	78.7 ± 133.1	
Median (min.–max.)	32.8 (1–650)	
Ritchie articular index [pts]		
Avg. ± SD	24.2 ± 17.7	
Median (min.–max.)	21.5 (1–53)	
Disease activity score 28 (DAS28)		
Me ± SD	3.8 ± 0.9	
Me (min.—max.)	4.0 (1.8–5.9)	
FACIT-F [pts.]		
Avg. ± SD	24.1 ± 9.1	
Median (min.–max.)	44.0 (7–49)	

Abbreviations: Avg., average; SD, standard deviation; min., minimum value; max., maximum value. FACIT-F scale—a maximum of 52 points can be obtained, indicating extreme fatigue, and a minimum of 0 points, indicating no fatigue. The range of points in the question is 0–4.

### 3.2 PSQI scale

The respondents scored from 0 to 17 on the PSQI scale. The average sleep quality in patients was 8.2 ± 4.0 points. Half of the patients scored no more than 8 points.

Most respondents had poor sleep quality (>5 points)—71.1% of women (*n* = 78). 27.3% (*n* = 30) of respondents had good sleep quality (≤5 points). The PSQI score could not be determined in the remaining patients, in 2 (1.6%) patients (because they could not provide all the data allowing the assessment of sleep quality in the questionnaire). Therefore, the remaining calculations will be performed for 108 women.

### 3.3 Relationships and correlations between demographic factors and the quality of sleep

Demographic factors such as gender, duration of the disease, education, place of residence, professional status, and residence status did not show a significant relationship with the quality of sleep on the PSQI scale (*p* > 0.05).

#### 3.3.1 Age and the quality of sleep

The t-test showed a significant difference in age level between patients with poor and good sleep quality (*p* = 0.0123). The average age was more important in people with poor sleep quality. Older women had worse sleep quality (Table 2).

**Table 2:** Descriptive statistics of age in women with poor and good sleep quality and t-test results.

PSQI	*n*	Age (year)					t	*p*
		Avg.	SD	Median	Min.	Maks.		
Poor sleep quality	78	55.8	13.8	58.0	23	83	2.54	0.0123*
Good sleep quality	30	48.6	15.5	51.0	19	74		

Abbreviations: Avg., average; SD, standard deviation; min., minimum value; max., maximum value; t, *t*-test value; p, probability level; *statistically significant, *p* < 0.05.

#### 3.3.2 Marital status and the quality of sleep

The Pearson chi-square test showed a significant relationship between patients’ marital status and sleep quality on the PSQI scale (*p* = 0.0367). Among married patients, there was a higher percentage of people with poor sleep quality (75%). Among unmarried patients, there was a higher percentage of people with good sleep quality (25%) (Table 3).

**Table 3:** Marital status in women with low and good sleep quality and the result of the Pearson chi-square test.

Marital status	PSQI				*χ* ^2^	df	*p*
	Poor sleep quality		Good sleep quality				
	*n*	%	*n*	%			
Living in a married relationship	66	81.5	21	77.8	4.36	1	0.0367*
Single	15	18.5	6	22.2			
Overall	81	100.0	27	100.0			

Abbreviations: df, degrees of freedom; *statistically significant, *p* < 0.05.

### 3.4 Relationships and correlations between clinical factors and the quality of sleep

Clinical factors such as pharmacological treatment, HB, CRP, rheumatoid factor RF, and articular index did not show a significant relationship with the quality of sleep on the PSQI scale (*p* > 0.05).

#### 3.4.1 Pain and the quality of sleep

The Mann–Whitney *U* test showed a significant difference in the VAS pain intensity level between patients with poor and good sleep quality (*p* = 0.0006). The mean and median VAS were higher in people with poor sleep quality. People experiencing more severe pain had poorer sleep quality. (Table 4).

**Table 4:** Descriptive statistics of VAS pain intensity in patients with poor and good sleep quality and the result of the Mann–Whitney *U* test.

PSQI	*n*	VAS					Z	*p*
		Avg.	SD	Median	Min.	Maks.		
poor sleep quality	78	6.1	2.4	6.0	0	10	3.42	0.0006*
good sleep quality	30	4.4	2.2	4.0	0	8		

Abbreviations: Avg., average; SD, standard deviation; min., minimum value; max., maximum value; *statistically significant, *p* < 0.05.

#### 3.4.2 Morning stiffness and the quality of sleep

The Mann–Whitney *U* test showed a significant difference in the level of morning stiffness between patients with poor sleep quality and patients with good sleep quality (*p* = 0.0002). Mean and median morning stiffness were higher in people with low sleep quality. Women who felt morning stiffness for more extended periods had worse sleep quality (Table 5).

**Table 5:** Descriptive statistics of morning stiffness in patients with poor and good sleep quality and the result of the Mann–Whitney U test.

PSQI	*n*	Morning stiffness (min)					Z	*p*
		Avg.	SD	Mediana	Min.	Maks.		
poor sleep quality	78	62.1	74.6	40.0	0	420	3.75	0.0002*
good sleep quality	30	28.3	46.0	10.0	0	240		

Abbreviations: Avg., average; SD, standard deviation; min., minimum value; max., maximum value; *statistically significant, *p* < 0.05.

#### 3.4.3 Disease activity DAS28 and the quality of sleep

The NW chi-square test showed a significant relationship between patient disease activity and sleep quality on the PSQI scale (*p* = 0.0367). Among patients with remission, the percentage of people with good sleep quality was much higher. Among patients with low disease activity, there was a higher percentage of people with poor sleep quality (18.5%). Among patients with moderate disease activity, the percentage with poor sleep quality was much higher (7.5%). Among patients with high disease activity, there was a higher percentage of people with good sleep quality (%) (Table 6).

**Table 6:** DAS28 disease activity in patients with poor and good sleep quality and the result of the NW chi-square test.

DAS28	PSQI				*χ* ^2^	df	*p*
	Poor sleep quality		good sleep quality				
	*n*	%	*n*	%			
remission	5	6,58	8	25	8.50	3	0.0367*
low disease activity	13	17,1	3	9,39			
average disease activity	53	69,74	18	56,26			
high disease activity	5	6,58	3	9,35			
Overall	76	100.0	32	100.0			

Abbreviations: df, degrees of freedom; *statistically significant, *p* < 0.05.

### 3.5 Correlation between sleep quality and level of fatigue

The Mann–Whitney *U* test showed a significant difference in the FACIT-F fatigue severity level between patients with poor and good sleep quality (*p* < 0.0001). The mean and median FACIT-F were higher in people with poor sleep quality. People with more significant fatigue had worse sleep quality (Table 7).

**Table 7:** Descriptive statistics of FACIT-F fatigue severity in patients with poor and good sleep quality and the result of the Mann–Whitney U test.

PSQI	*n*	FACIT-F (pct)					Z	*p*
		Avg.	SD	Median	Min.	Maks.		
poor sleep quality	78	26.2	8.8	26.0	0	49	4.73	<0.0001*
good sleep quality	30	17.7	7.7	16.0	8	37		

Abbreviations: Avg., average; SD, standard deviation; min., minimum value; max., maximum value; *statistically significant, *p* < 0.05.

### 3.6 Multiple regression

Table 8 presents the results of multiple regression analysis for the quality of life variable and five models of independent variables:

(1) Model 1 analyzed the impact of hemoglobin, CRP, and RF on sleep quality—the regression model was statistically insignificant.
(2) Model 2 assessed the combined effect of treatment (biological/conventional drugs) and pain on sleep quality—the regression model turned out to be statistically significant (*p* < 0.0001), and the regression model itself explains 15.5% of the variability in PAQI sleep quality. Increasing pain intensity by one unit increases the PSQI score (decreased sleep quality) by an average of 0.657 ± 0.139 points, with the remaining variable remaining constant.
(3) Model 3 analyzed the impact of pain and DAS28 disease activity on the level of sleep quality—the regression model turned out to be statistically significant (*p* < 0.0001), and the regression model explains 16.2% of the variability of PAQI sleep quality. Increasing pain intensity by one unit increases the PSQI score (decreased sleep quality) by an average of 0.654 ± 0.148 points, with the remaining variable remaining constant.
(4) Model 4 assessed the combined effect of morning stiffness, Articular index, and pain on the level of sleep quality—the regression model turned out to be statistically significant (*p* < 0.0001), and the regression model itself explains 16.2% of the variability of PAQI sleep quality. Increasing pain intensity by one unit increases the PSQI score (decreased sleep quality) by an average of 0.723 ± 0.156 points, with other variables remaining constant.
(5) Model 5—analyzed the impact of morning stiffness, DAS28 disease activity, and CRP on the level of sleep quality—the regression model was statistically insignificant (*p* > 0.05).

**Table 8:** Multiple regression results for the PSQI variable and independent variables.

Variables	b*	Std. error for b*	b	bp	*p*
Model 1					
R = 0.039; *R*^2^ = 0.002;					
F(3.122) = 0.06; *p* = 0.9794; Błąd std. estymacji: 4.09					
Hb [g/dl]	−0.006	0.093	−0.008	0.135	0.9505
CRP [mg/L]	0.009	0.099	0.002	0.026	0.9259
RF [IU/ml]	0.034	0.097	0.001	0.003	0.7283
Model 2					
R = 0.393; *R*^2^ = 0.155; Popraw. *R*^2^ = 0.141;					
F(3.123) = 11.24; *p* < 0.0001; Błąd std. estymacji: 3.75					
Pharmacological treatment	0.064	0.084	0.653	0.858	0.4480
VAS [cm]	0.397	0.084	0.657	0.139	<0.0001
Model 3					
R = 0.388; *R*^2^ = 0.151; Popraw. *R*^2^ = 0.137;					
F(3.123) = 10.93; *p* < 0.0001; Błąd std. estymacji: 3.75					
VAS [cm]	0.395	0.089	0.654	0.148	<0.0001
DAS28	−0.019	0.089	−0.082	0.389	0.8333
Model 4					
R = 0.403; *R*^2^ = 0.162; Popraw. *R*^2^ = 0.142;					
F(3.122) = 7.89; *p* < 0.0001; Błąd std. estymacji: 3.74					
VAS [cm]	0.437	0.094	0.723	0.156	<0.0001
Morning stiffness [min]	−0.021	0.092	−0.001	0.005	0.8192
Articular index [pkt]	−0.111	0.090	−0.025	0.020	0.2189
Model 5					
R = 0.403; *R*^2^ = 0.162; Popraw. *R*^2^ = 0.142;					
F(3.122) = 7.89; *p* < 0.0001; Błąd std. estymacji: 3.74					
Morning stiffness [min]	0.126	0.104	0.007	0.006	0.2293
CRP [mg/L]	−0.104	0.111	−0.027	0.029	0.3498
DAS28	0.131	0.104	0.572	0.451	0.2069

R, correlation coefficient; *R*^2^, correlation of determination; F, *F* test statistic.

### 3.7 Logistic regression

The same factor models, as in the regression analysis, were subjected to logistic analysis:

(1) Model 1 analyzed the impact of hemoglobin, CRP, and RF on sleep quality—the regression model was statistically insignificant (*p* > 0.05).
(2) Model 2 assessed the combined effect of treatment (biological/conventional drugs) and pain on sleep quality—the regression model turned out to be statistically significant (*p* = 0.0019), and the quality of model fit is low [Pseudo R2 = 0.084; R2(Nagelkerke) = 0.136; R2(Cox- Snell) = 0.094]. For PSQI, only the VAS pain intensity was a significant variable (*p* = 0.0011). The higher the pain intensity, the lower the chance of good-quality sleep.
(3) Model 3 analyzed the impact of pain and DAS28 disease activity on the level of sleep quality
  1. The regression model turned out to be statistically significant (*p* = 0.0008), and the quality of model fit is not high [Pseudo R2 = 0.127; R2(Nagelkerke) = 0.201; R2 (Cox-Snell) = 0.139]. The following variables turned out to be essential for PSQI:
  2. VAS pain intensity (*p* = 0.0023)—the higher the pain intensity, the lower the chance of good quality sleep,
  3. DAS28 (*p* = 0.0469)—in people with low disease activity, the chance of good sleep quality is almost 5.3 times lower than in people with remission.
(4) Model 4 assessed the total impact of morning stiffness, Articular index, and pain on the level of sleep quality—the regression model turned out to be statistically significant (*p* = 0.0002), and the quality of model fit is not high [Pseudo R2 = 0.131; R2(Nagelkerke) = 0.206; R2(Cox- Snell) = 0.143]. For PSQI, only the VAS pain intensity was a significant variable (*p* = 0.0048). The higher the pain intensity, the lower the chance of good-quality sleep.
(5) Model 5—analyzed the impact of pain, hemoglobin, and treatment (biological/conventional drugs) on the level of sleep quality—the logistic regression model turned out to be significant (*p* = 0.0004). The remaining variables turned out to be statistically insignificant (*p* > 0.05). The quality of the model fit is not high: pseudo R2 = 0.139, R2(Nagelkerke) = 0.221, R2(Cox- Snell) = 0.156. The significant parameter was the VAS variable (*p* = 0.0002). The higher the VAS pain level, the lower the chance of good sleep in women (with the remaining variables at a constant level).
(6) Model 6—analyzed the impact of morning stiffness, DAS28 disease activity, and CRP together on the level of sleep quality—the regression model turned out to be statistically significant (*p* = 0.0008), and the quality of model fit is not high [Pseudo R2 = 0.121; R2(Nagelkerke)] = 0.193; R2(Cox-Snell) = 0.133). The following variables turned out to be essential for PSQI:
  4. morning stiffness (*p* = 0.0165)—the longer the morning stiffness, the lower the chance of good quality sleep,
  5. DAS28 (2) (*p* = 0.0250)—in people with low disease activity, the chance of good sleep quality is almost 6.3 times lower than in people with remission.

## 4 Discussion

Assessment of sleep quality in patients with various diseases is essential in their holistic care. As sleep affects all life processes, the intensity of disease symptoms, and the patient's well- being, it is necessary to consider the patient's individual conditions during therapy.

### 4.1 Sleep quality

Our research has shown that women with rheumatic disease have low sleep quality (71.1%). Most of the scientific reports to date also confirm these results. Szady et al. (7) also confirmed poor sleep quality among Polish RA female patients (63.16% of 38 patients). Worse sleep quality and the occurrence of insomnia in RA patients were also indicated by Mena-Vázquez et al. (15). Similar results were obtained by Kontodimopoulos et al. (16) and McBeth et al. (17). In the study by Genta et al. (18), as many as 95% of patients indicated poor sleep quality. Durcan et al. also demonstrated poorer sleep quality among RA patients (19). It is worth emphasizing that the abnormal sleep quality was influenced by factors such as difficulty falling asleep, frequent waking up, and too little sleep. Different results were obtained by Miyauchu et al. (20), where the sleep quality did not differ statistically significantly between Japanese women with RA and the control group. In the study by Goes et al. (21), only 20% of 112 patients with RA had good quality sleep. 96 RA patients (out of 187) in the study by Guaracha-Basáñez et al. (22) had optimal sleep. Poor sleep quality among Mexican patients (41.75% of 102 subjects) was observed by Juárez-Rojop et al. (23). Lyne et al. (24), in a cohort study of 4,131 RA patients, obtained the results that 39% of the subjects had problems with sleep, at least in one of the domains of the Karolinska Sleep Questionnaire (sleep problems, non-restorative sleep, insomnia, insufficient sleep, sleep quality perceived as poor and sleep considered a health problem).

### 4.2 Sleep quality and fatigue

Our research showed that a high level of fatigue is correlated with poor sleep quality in RA patients. This was confirmed in their studies by Szady et al. (7), Gouda et al. (5), McBeth et al. (17), Durcan et al. (19), Guaracha-Basáñez et al. (22), Austad et al. (25) and Shen et al. (26), Kontodimopoulos et al. (16), Katz et al. (27, 28), Løppenthin et al. (29), Hammam et al. (30). Genty et al. (18), analyzing the relationship between fatigue and sleep quality, showed that improving sleep quality does not change the level of fatigue in RA patients. Goes et al. (21) also linked fatigue with poor sleep quality, emphasizing that fatigue correlates with pain (9), which worsens sleep.

The study's authors obtained research results indicating that poorer sleep quality is associated with older age, marital status (married people), high levels of fatigue, severe pain, more prolonged morning stiffness, and disease activity (low and medium disease activity).

### 4.3 Sleep quality and pain

The influence of pain on the deterioration of sleep quality was also confirmed by Szady et al. (7). They also proved that older age and marital status also influence poor sleep. Austad et al. (25) indicated a moderate correlation between pain and disease activity and sleep quality in their research. Pain was also stated as the main predictor of poorer sleep quality by Gouda et al. (5), McBeth et al. (17), Durcan et al. (19), Guaracha-Basáñez et al. (22), and Shen et al. ( 26). The influence of age and disease activity was confirmed by Mena-Vázquez et al. (15), indicating that comorbidities also negatively affect the quality of sleep, with particular emphasis on interstitial lung disease. The negative impact of disease activity on sleep quality was confirmed by Kontodimopoulos et al. (16). Katz et al. (32) also showed in their research that the active form of the disease and pain worsen sleep quality. The impact of disease activity on the deterioration of sleep quality was also noted by Goes et al. (21), who link the effects of these factors with pain because the active form of the disease is associated with the feeling of pain. Among Turkish subjects, pain and the age of spouses influenced sleep quality—the older the subjects were, the lower their sleep quality (32). Deniz et al. (33) confirmed that older age and disease activity are factors that worsen sleep quality. The influence of age on sleep quality was not confirmed by Gouda et al. (5) and McBeth et al. (17). Married people had better sleep quality than single people among Mexican respondents (22). Studies by Gouda et al. (5) and Mustafa et al. (34) showed that disease activity does not significantly impact sleep quality.

### 4.4 Sleep quality and psyche

It is worth noting that many research authors indicate that the level of sleep quality is also influenced by factors such as depression, anxiety, mood, physical fitness, level of disability, practicing sports, relaxation, comorbidities or sleep disorders resulting from physiological reasons (e.g., sleep apnea, shortness of breath), which were not analyzed in this study (5, 21–29). Interesting research was conducted by Gao et al. (35), who examined the influence of sleep characteristics of the causation of RA. The assessment included short sleep duration, frequent insomnia, and insomnia, sleep duration, getting up, morningness (early-to-bed/up habit), and snoring. The obtained results indicated that RA is influenced only by short sleep, less than or equal to 6 h.

*According to our research, sleep quality is not associated with BMI, disease duration, education, place of residence, professional status, residence status, pharmacological treatment, HB, CRP and RF levels, or Articular index*.

Similar results were obtained by Mcbeth et al. (17), where marital status, and pharmacological treatment did not affect the quality of sleep, and in the study by Gouda et al. (5) also, the CRP level and the disease duration. The type of pharmacological treatment was not significantly associated with sleep quality, as also in Hughes et al. (36). However, it was indicated that steroids significantly worsen sleep quality compared to other types of treatment. A study by Lyne et al. (24) showed no relationship between the duration of the disease and the quality of sleep.

Different data were obtained by Szady et al. (7), who indicated in their research that women experience poorer sleep quality than men and that pharmacological treatment and a longer duration of the disease influence its level. The influence of gender on sleep quality was confirmed by Lyne et al. (24). In the study by Goes et al. (21), pharmacological treatment with prednisone reduced sleep quality in RA patients. The BMI index was indicated as one of the predictors indicating whether the patient will have good or poor sleep quality by Guaracha- Basáñez et al. (22). The number of affected and tender joints was positively correlated with the level of sleep quality by Pehlivan et al. (33) and Hughes et al. (36), as opposed to swollen joints (29).

Studies by Chen et al. (37), Demir et al. (38), Rubbert-Roth et al. (39), and Padjen et al. (40) indicate that low hemoglobin level (anemia) is one of the factors that affect RA, either disease activity or indirectly, sleep quality/disorders. The women examined in this study showed features of anemia (Avg. ± SD—9.3 ± 2.8); however, the low hemoglobin level itself did not significantly affect the level of sleep quality. However, it may have indirectly influenced other parameters, such as fatigue or disease activity, which considerably affected sleep quality. It is worth noting that the low haemoglobin values presented by the examined patients are quite atypical. This study aimed not to analyze the cause of the occurrence of low values and the method of treating anaemia. However, based on the studies of other authors, it can be interpreted that low haemoglobin values may result from the activity of the disease (71 women DAS28—average disease activity), the degree of joint damage (Ritchie Articular Index—Avg. 23.6) (37, 40) and even the treatment used (only 19 women were treated biologically) (39).

### 4.5 Multiple and logistic regression

Based on our research using multivariate analysis, it was indicated that the only determinant that reduces sleep quality is the pain experienced by RA patients. Linear regression showed that pain, low disease activity (together), and longer-lasting morning stiffness and low disease activity (together) strongly impacted sleep quality.

Using logistic regression, Grabovac et al. (6) confirmed that pain was the only component significantly affecting sleep quality, which was not demonstrated for age. In a multivariate analysis conducted by Mena-Vázquez et al. (15), the influence of age, comorbid interstitial lung disease, and DAS28 on sleep quality was examined. Only age and disease activity were found to be these factors. They influenced. Juárez-Rojop et al. (23) assessed whether hip joint involvement, anxiety symptoms, marital status, and depression jointly affect the level of sleep quality. Depression and drug-related conditions were essential factors. However, in a linear multivariate analysis conducted by Swedish researchers Løppenthin et al. (29), fatigue (general and mental) was a factor influencing the deterioration of sleep quality.

### 4.6 Limitations of the study

It should be noted that the results of our own research are associated with certain limitations resulting from the adopted assumptions and methodology. No studies were conducted on the repeatability of results in the same patients, especially patients in the period of active disease and then remission, after a change in pharmacological treatment or its implementation. The influence of comorbidities was not analyzed, and thus the methods of their treatment. The authors of the paper are also aware of the factors that could have influenced the patients when assessing the level of sleep quality and fatigue—such as stress, anemia, mental condition, haste in providing answers, underestimated/imprecise answers. However, this allows for further, in-depth studies, with the analysis of a larger number of factors that may directly or indirectly affect the quality of sleep in RA patients.

## 5 Conclusions

RA women have worse sleep quality, which may be influenced by demographic factors (such as age or marital status), clinical factors (morning stiffness, pain, disease activity), and fatigue. Analyzing the available literature, the discrepancies in the results are considerable. Therefore, it is worth continuing research on larger groups of patients and considering more factors that may worsen or improve sleep quality.

It is also an indication for doctors that a holistic approach to a patient can significantly improve his quality of life, and improving one factor will optimally influence others. A change in the intensity or complete elimination of one of the symptoms may affect the well-being of a RA patient.

## Data Availability

The original contributions presented in the study are included in the article/Supplementary Material, further inquiries can be directed to the corresponding author.
